# Widening health inequalities between the employed and the unemployed: A decomposition of trends in Canada (2000-2014)

**DOI:** 10.1371/journal.pone.0208444

**Published:** 2018-11-29

**Authors:** Faraz Vahid Shahidi, Carles Muntaner, Ketan Shankardass, Carlos Quiñonez, Arjumand Siddiqi

**Affiliations:** 1 Dalla Lana School of Public Health, University of Toronto, Toronto, Ontario, Canada; 2 Bloomberg School of Nursing, University of Toronto, Toronto, Ontario, Canada; 3 Department of Health Sciences, Wilfrid Laurier University, Waterloo, Ontario, Canada; 4 Faculty of Dentistry, University of Toronto, Toronto, Ontario, Canada; 5 Gillings School of Public Health, University of Northern Carolina, Chapel Hill, North Carolina, United States of America; University Complutense of Madrid, SPAIN

## Abstract

Recent developments in the social epidemiological literature indicate that health inequalities between the employed and the unemployed are widening in many advanced capitalist countries. At present, we know relatively little about why these inequalities are worsening. Drawing on nationally-representative data from the largest health survey in Canada, we explored this question by analyzing changes in self-rated health inequalities between employed and unemployed Canadians from 2000 to 2014. Using a regression-based method that decomposes a given inequality into its component sources, we investigated the extent to which risk factors that account for unemployment-related health inequalities at a single point in time can also explain the extent and direction of change in these unemployment-related health inequalities over time. Our results indicate that relative and absolute health inequalities between employed and unemployed Canadians widened over the study period. Between 2000 and 2014, the prevalence of poor self-rated health among unemployed Canadians increased from 10.8% to 14.6%, while rates among employed Canadians were stable at around 6%. Our findings suggest that the demographic, socioeconomic, and proximal risk factors that are routinely used to explain unemployment-related health inequalities may not be as powerful for explaining how and why these inequalities change over time. In the case of unemployment-related health inequalities in Canada, these risk factors explain neither the increasing prevalence of poor self-rated health among the unemployed nor the growing gap between the unemployed and their employed counterparts. We provide several possible explanations for these puzzling findings. We conclude by suggesting that widening health inequalities may be driven by macrosocial trends (e.g. widening income inequality and declining social safety nets) which have changed the meaning and context of unemployment, as well as its associated risk factors, in ways that are not easy to capture using routinely available survey data.

## Introduction

Over the past several decades, scholars have dedicated a large and rapidly expanding body of scientific literature to the study of health inequalities, by which we mean systematic differences in the health of populations and population groups [1–3]. Despite significant advances in our scientific understanding of this pressing problem, recent developments in the literature indicate that little progress has been made towards the goal of reducing health inequalities [4,5]. In fact, a growing body of evidence suggests that health inequalities between socioeconomic groups are widening in many advanced capitalist countries [6–10].

In this paper, we narrow in on the specific case of widening unemployment-related health inequalities and assess possible explanations for these troubling epidemiological trends. Unlike income and education, employment status and other indicators of labour market position have been awarded relatively scant attention in the health inequalities literature, despite their importance as major determinants of health [11]. Notwithstanding this limitation of the literature, recent findings suggest that relative and absolute health inequalities between the employed and the unemployed are widening over time [12–14]. While the reasons for this trend are not well understood, extant research points to several potential hypotheses, which we review below [15]. A summary of these hypotheses and their corresponding literatures is provided in Table 1.

**Table 1:** Summary of hypotheses to help explain widening unemployment-related health inequalities.

Hypothesis	Explanation	Underlying Literature
Mathematical Artifact	Evidence of widening unemployment-related health inequalities is an artifact of the general mathematical tendency for relative inequalities to increase following declines in the overall frequency of an outcome.	Eikemo et al. [16]; Houweling et al. [17]; Huijts and Eikemo [18]; Mackenbach [19]; Scanlan [20]
Indirect Social Selection	Due to an increasing scope for social selection on traits such as intelligence and cognitive ability, the unemployed consist of an increasingly negatively selected group of individuals, resulting in widening unemployment-related health inequalities.	Boyle et al. [22]; Dowd and Hamoudi [23]; Hughes et al. [24]; Jusot et al [25]; Lundin et al. [26]; Mackenbach [4]; Steele et al. [27]; Tøge and Blekesaune [28]
Proximal Risk Factors	Widening unemployment-related health inequalities are a product of widening inequalities in the distribution of proximal risk factors (e.g. smoking, drinking, and physical inactivity) between the employed and the unemployed.	Deb et al [31]; Kalousova and Burgard [32]; Macy et al. [33]; Marcus [34]; Monsivais et al. [34]; Mossakowski [36]; Schunck and Rogge [37]; Virtanen et al. [38]
Social Conditions	Widening unemployment-related health inequalities are a product of widening inequalities in the distribution of key socioeconomic resources (e.g. income, education, and wealth) between the employed and the unemployed.	Brydsten et al. [44]; Córdoba-Doña et al. [49]; Farrants et al. [12]; Huijts et al. [45]; Kroll and Lampert [13]; McCartney et al. [51]; McLeod et al. [50]; Nelson and Tøge [14]; Price et al. [46]; Riumallo-Herl et al. [47]; Tøge [48]

A first hypothesis posits that unemployment-related health inequalities may be widening due to the general tendency for relative inequalities to grow as the absolute prevalence of an outcome declines [16–20]. According to this view of the problem, widening health inequalities between the employed and the unemployed may be a *mathematical artifact* of underlying improvements in the overall health of the general working-age population. Though such a view raises important questions concerning the choice of absolute or relative indicators in the measurement of health inequality [21], it falls short of explaining why both relative and absolute health inequalities have increased between the employed and the unemployed.

A second hypothesis suggests that *indirect social selection* may be a key factor contributing to the evolution of unemployment-related health inequalities [22–28]. The notion here is that, as societies become more socially mobile, personal characteristics such as intelligence and cognitive ability can play an increasing role in shaping socioeconomic outcomes, including those pertaining to the labour market [4]. From this point of view, the growing health gap between employed and unemployed workers may reflect an increasing scope for indirect selection on the basis of these personal traits. In other words, the unemployed today may represent a more negatively selected group of workers than at earlier points in time. Notably, this argument is premised on the assumption that advanced capitalist societies have become more meritocratic over time. However, recent findings indicate that rates of social mobility have remained stable or, worse, declined in recent decades [29,30].

Another hypothesis addresses the potential contribution of *proximal risk factors* such as smoking, drinking, physical inactivity, obesity, and stress to the changing magnitude of health inequalities between the employed and the unemployed [31–38]. Recent epidemiologic studies suggest that these proximal risk factors account for more than half of the health inequalities observed between major socioeconomic groups [39–41]. Changes in the magnitude of unemployment-related health inequalities may therefore reflect changes in the patterning of proximal risk factors between the employed and the unemployed. In other words, relative to earlier points in time, the unemployed today may exhibit a worse set of proximal risk factors relative to their employed counterparts, thereby contributing to a widening health gap between these two groups. Indeed, while evidence pertaining specifically to the unemployed is currently lacking, findings from the broader literature suggest that inequalities in proximal risk factors between other key socioeconomic (e.g. income and education) groups have been widening [42,43].

The fourth and final hypothesis stresses the importance of *social conditions* as fundamental causes of unemployment-related health inequalities [12–14,44–50]. Those who adopt such a view suggest that widening inequalities between the employed and the unemployed are a predictable consequence of widening inequalities in the distribution of key resources such as income and wealth [51]. Indeed, over the past several decades, we have witnessed a steep increase in the magnitude of economic inequality in nearly all advanced capitalist countries [52]. This growing economic wedge appears to be driven by underlying changes in the structure of the labour market, including an increasing prevalence of precarious employment conditions, rising levels of structural unemployment, stagnating earnings among low-wage workers, and the enactment of wide-ranging labour market reforms that have curtailed the scope and generosity of redistributive social policies [53,54]. Put simply, these macrosocial trends have widened socioeconomic inequalities, such as those observed between the employed and the unemployed. This may in turn explain why unemployment-related health inequalities between have been widening over time.

In this paper, we adjudicate between these hypotheses by exploring changing patterns of unemployment-related health inequality in Canada. We use nationally representative repeated cross-sectional data from the Canadian Community Health Survey (CCHS) to analyze and explain trends in the self-rated health of employed and unemployed Canadians between 2000 and 2014. Specifically, we use a counterfactual method known as decomposition to investigate whether and to what extent a range of demographic, socioeconomic, and proximal risk factors account for (i) change over time in the self-rated health of unemployed Canadians and (ii) change over time in the magnitude of self-rated health inequalities between employed and unemployed Canadians. We approach the issue in this manner because the factors determining change *within* a socioeconomically disadvantaged group may differ from the factors determining change *between* that group and a more socioeconomically advantaged counterpart group.

Based on this approach, we are able to answer the following questions:

1. How would the health status of the unemployed in 2013/2014 have differed had they been endowed with the demographic, socioeconomic, and proximal characteristics of their unemployed counterparts in 2000/2001?
2. How would the health status of the unemployed have differed at each point in time had they been endowed with the demographic, socioeconomic, and proximal characteristics of their employed counterparts?

## Materials and methods

### Data

The CCHS is a repeated cross-sectional survey containing nationally representative data on the health of Canadians above the age of 12. The first cycle was administered in 2000/2001. Cycles were administered biennially until 2005 and annually from 2007 onwards. Our study covered the period from 2000 to 2014. We did not include more recent cycles due to the implementation of a major redesign in 2015. The biennial cycles included approximately 130,000 observations each, while the annual cycles included approximately 65,000 observations each. To establish similar sample sizes and a consistent unit of time, we grouped annual cycles into pairs. This resulted in seven time points corresponding respectively to the following years: 2000/2001, 2003, 2005, 2007/2008, 2009/2010, 2011/2012, and 2013/2014.

### Sample

The sample included individuals who were between the ages of 18 and 64 and either employed full-time (*i*.*e*. 30 or more hours per week) or unemployed and actively seeking work at the time of survey administration. Part-time workers, students, and individuals who were jobless but not actively seeking work (e.g. full-time caregivers, early retirees, discouraged workers, and those permanently unable to work) were excluded from the analysis. We also excluded residents of the northern territories (*i*.*e*. Yukon, Northwest Territories, and Nunavut) for whom equivalized household income data could not be collected. The final sample consisted of 337,880 individuals, of which 318,245 were employed full-time and 19,635 were unemployed.

### Outcome variable

The outcome of interest was a dichotomous measure of self-rated health, widely considered to be a valid and reliable predictor of morbidity and mortality [55]. We measured self-rated health using a single five-category variable that asked respondents to rate their general health. The variable distinguished between individuals who reported good (“excellent”, “very good”, or “good”) and poor (“fair” or “poor”) self-rated health.

### Predictor variables

We included three groups of predictors, representing demographic, socioeconomic, and proximal determinants of poor self-rated health. We provide a summary and description of these variables in Table 2. Demographic factors included age (years), sex (male versus female), marital status (couple, single, or widowed/divorced), whether any children live in the household, self-identified race (white, black, Aboriginal, Asian, or multiple/other), immigrant status (non immigrant, immigrant in Canada less than 15 years, or immigrant in Canada for 15 years or more), region (Atlantic, Central, or Western), and urbanicity (urban versus rural).

**Table 2:** Description of study variables.

Variable Name	Description
Self-Rated Health	Dummy Variable; 0 = good, very good, or excellent; 1 = fair, or poor
Age	Ordinal Variable; 1 = 18–24; 2 = 25–34; 3 = 35–44; 4 = 45–54; 5 = 55–64
Sex	Dummy Variable; 0 = male; 1 = female
Marital Status	Nominal Variable; 1 = couple; 2 = single; 3 = widowed or divorced; 4 = missing
Children	Dummy Variable; 0 = no children; 1 = one or more children; 3 = missing
Self-Reported Race or Ethnicity	Nominal Variable; 1 = white; 2 = black; 3 = Aboriginal; 4 = Asian; 5 = multiple or other; 6 = missing
Immigrant Status	Nominal Variable; 1 = not an immigrant; 2 = an immigrant in Canada less than 15 years; 3 = an immigrant in Canada for 15 years or more; 4 = missing
Region	Nominal Variable; 1 = Atlantic Canada (New Brunswick, Newfoundland and Labrador, Nova Scotia, Prince Edward Island); 2 = Central Canada (Ontario, Quebec); 3 = Western Canada (British Columbia, Alberta, Saskatchewan, Manitoba)
Urban/Rural	Dummy Variable; 0 = urban; 1 = rural
Education	Ordinal Variable; 1 = less than a secondary degree; 2 = secondary degree; 3 = some post-secondary education; 4 = post-secondary degree; 5 = missing
Income	Ordinal Variable: 1 = first decile; 2 = second decile; 3 = third decile; 4 = fourth decile; 5 = fifth decile; 6 = sixth decile; 7 = seventh decile; 8 = eighth decile; 9 = ninth decile; 10 = tenth decile; 11 = missing
Home Ownership	Nominal Variable; 1 = owns their own; 2 = rents their home; 3 = missing
Employment Insurance	Nominal Variable; 1 = collected employment insurance this year; 2 = did not collect employment insurance this year; 3 = missing
Social Assistance	Nominal Variable; 1 = collected social assistance this year; 2 = did not collect social assistance this year; 3 = missing
Self-Rated Stress	Nominal Variable; 1 = a bit, not very, or not at all stressed; 2 = quite a bit or extremely stressed; 3 = missing
Chronic Conditions	Nominal Variable; 1 = diagnosed at least once with either asthma, chronic bronchitis, heart disease, cancer, diabetes, stroke, or Alzheimer’s disease; 2 = never diagnosed with asthma, chronic bronchitis, heart disease, cancer, diabetes, stroke, and Alzheimer’s disease; 3 = missing
Hypertension	Nominal Variable; 1 = diagnosed at least once with hypertension; 2 = never diagnosed with hypertension; 3 = missing
Obesity	Nominal Variable; 1 = body mass index of less than 30; 2 = body mass index of 30 or more; 3 = missing
Smoking	Nominal Variable; 1 = never smoked; 2 = former smoker; 3 = current smoker; 4 = missing
Drinking	Nominal Variable; 1 = non-drinker; 2 = moderate drinker; 3 = binge drinker; 4 = missing
Physical Activity	Nominal Variable; 1 = sufficiently active; 2 = somewhat active; 3 = inactive; 4 = missing

Socioeconomic factors included education (less than secondary, secondary degree, some post-secondary, or post-secondary degree), home ownership (renter versus owner), household income (decile), and, among the unemployed, household receipt of social assistance or federal unemployment benefits. To account for household size in the measurement of income, we used a method of equivalization adopted in recent OECD publications that involves dividing household income by the square root of the household size [56].

Proximal risk factors included self-rated stress, chronic conditions, hypertension, obesity, smoking, binge drinking, and physical inactivity. We measured self-rated stress using a single five-category question that asked respondents to rate overall levels of stress in their life. The variable distinguished between those who reported low (“a bit”, “not very”, or “not at all”) and high (“quite a bit” or “extremely”) levels of stress. A dichotomous variable identified whether respondents had ever been diagnosed with one or more of the following chronic conditions: asthma, chronic bronchitis, heart disease, cancer, diabetes, stroke, or Alzheimer’s disease. We selected these conditions because they are listed among the leading causes of death in Canada [57]. A separate dichotomous variable identified whether respondents had ever been diagnosed with hypertension. Obesity was defined as a body mass index of 30 or above, using self-reported height and weight variables. Health behaviours included smoking (non-smoker, former smoker, or current smoker), drinking (non-drinker, current moderate drinker, current binge drinker), and physical activity (sufficiently active, somewhat active, inactive). Following Statistics Canada practice, we defined binge drinking as the consumption of five or more standardized alcoholic drinks on one occasion, twelve or more times over the past year. We measured the sufficiency of physical activity using a derived index variable based on daily activities over the past three months. Though the CCHS includes some questions about adverse psychosocial experiences, dietary behaviours, and food insecurity, they were situated in optional content modules that several provinces chose not to include. As a result, they could not be included in our study.

### Statistical analysis

We used weighted proportions to describe the demographic, socioeconomic, and proximal characteristics of the sample. We provided separate descriptives for employed and unemployed individuals at each time point. For each group, we also plotted unadjusted trends in the prevalence of poor self-rated health over the duration of the study period. Following this descriptive analysis, we performed a decomposition analysis to investigate candidate explanations for (i) change over time in the self-rated health of the unemployed individuals and (ii) change over time in the magnitude of self-rated health inequalities between employed and unemployed individuals.

Decomposition refers to the use of statistical methods to examine the determinants of inequalities [58,59]. Decomposition methods draw on a suite of regression-based techniques to estimate the contribution of specific predictors (or sets of predictors) to a given inequality. Through an evaluation of counterfactuals in which one group is endowed with the characteristics of another, these methods quantify the portion of the inequality that is attributable to differences in the distribution of those characteristics. Relying on this counterfactual approach—also known as the potential outcomes framework—decomposition methods broaden the scope of questions we can ask about inequalities and their underlying causes, beyond those amenable to standard regression methods [60–63]. In the present study, the use of decomposition methods allows us to answer the counterfactual questions we posed in our introduction; namely (i) “How would the health status of the unemployed in 2013/2014 have differed had they exhibited the same predictor profile as their unemployed counterparts in 2000/2001?”, and (ii) “How would the health status of the unemployed have differed had they exhibited the same predictor profile as their employed counterparts at each point in time?” Whereas standard regression techniques (e.g. logistic regression) would be most suitable for examining how relative measures of risk associated with (i) time and (ii) employment status respond as sets of predictors are added to a given regression model, decomposition methods instead enable us to estimate the absolute reduction of risk that would result from the counterfactual elimination of inequalities in each specific predictor. Following earlier debate over the value of counterfactual thinking in the public health sciences [64–67], decomposition methods have recently gained significant prominence as a powerful tool with which to identify the underlying causes of health inequalities between groups [44,68–76], as well as the evolution of health outcomes in the same group or population [77–81].

The most common decomposition method is the Oaxaca-Blinder reweighting procedure, originally used to examine the causes of wage inequalities [82,83]. This procedure was designed for linear outcome models. Because our outcome was binary, we instead used a non-linear extension of the method developed by Fairlie [84]. Following Fairlie, we define the non-linear decomposition of an inequality between reference group *R* and comparison group *C* as follows:

$${\overset{-}{Y}}^{R}–{\overset{-}{Y}}^{C}=\left[\sum_{i=1}^{N^{R}}\frac{F(X_{i}^{R}{\hat{B}}^{R})}{N^{R}}-\sum_{i=1}^{C}\frac{F(X_{i}^{C}{\hat{B}}^{R})}{N^{C}}\right]+\left[\sum_{i=1}^{N^{C}}\frac{F(X_{i}^{C}{\hat{B}}^{R})}{N^{C}}-\sum_{i=1}^{N^{C}}\frac{F(X_{i}^{C}{\hat{B}}^{C})}{N^{C}}\right]$$

where $\bar{\text{Y}}$ refers to the average value of an outcome, *X* refers to the average value of a vector of predictors, $\hat{\text{B}}$ refers to a vector of coefficient estimates, and *N* refers to sample size. As shown in the above equation, the non-linear decomposition of a function produces two terms. The first term represents the portion of the difference that is attributable to group differences in the distribution of observed characteristics. The second term captures the portion of the difference that is left unexplained after the comparison group is endowed with the characteristics of the reference group. We refer to these as the endowment and residual terms, respectively. Residual terms arise when there are either unmeasured sources of variation or group differences in the effects of measured characteristics.

We obtained all estimates using sampling weights provided by Statistics Canada. To derive reliable standard errors, we averaged our decomposition results across 1000 repeated bootstrap samples. Decomposition results can depend heavily on the conditional order in which predictors are entered. For this reason, we ordered predictors randomly across the repeated samples. We conducted all analyses using Stata 13.0 (StataCorp LP, College Station, TX).

### Missing values

We dropped observations missing information on labour market position or self-rated health. This amounted to less than 1% of the original sample. We tested the equivalence of the samples before and after dropping these observations and found no statistically significant differences across all variables (p<0.05). However, sensitivity analyses revealed that dropping observations missing one or more predictor value introduced substantial bias to trends in our outcome variable. We therefore adopted a missing indicator approach and included these observations in our analysis. For applications of this approach in the decomposition literature, see Fairlie and Robb [85] and Lin and colleagues [86]. Notably, the proportion of observations in any given missing category tended to be very small (*i*.*e*. less than 2%). A key exception to this was the high proportion of observations in the first two cycles of the CCHS with missing household income values. From 2005 onwards, all missing household income values were imputed by Statistics Canada. We consider the implications of this missing information in our discussion of the results.

### Ethics statement

Data for the Canadian Community Health Survey are collected and maintained by Statistics Canada. All data were fully anonymized before being accessed. Participation in the survey is voluntary and respondents are asked to provide written informed consent prior to participation. The study was approved by the University of Toronto Research Ethics Board.”

## Results

### Descriptive characteristics

We present the demographic, socioeconomic, and proximal characteristics of the sample at each time point in Tables 3–5. Relative to their employed counterparts, unemployed individuals were younger, more likely to be single, and less likely to be white. Unemployed individuals reported lower levels of household income, educational attainment, and home ownership. For example, the proportion of respondents in 2013/2014 who reported household income levels in the highest income decile was 4.9% among the unemployed and 14.3% among the employed. In the same year, the proportion of respondents who reported owning their home was 52.8% among the unemployed and 74.5% among the employed. Unemployed individuals reported consistently higher rates of chronic conditions and smoking but lower rates of drinking and physical inactivity. Both groups experienced similar compositional changes over time. Notable trends included a rightward shift in the distribution of age, an increasing proportion of racialized minorities and immigrants, and increasing rates of educational attainment. For example, between 2000/2001 and 2013/2014, the proportion of respondents with less than a high school degree decreased from 25.0% to 14.2% among the unemployed and from 14.6% to 7.0% among the employed. Both groups reported increasing rates of obesity and hypertension as well as declining rates of smoking and physical inactivity.

**Table 3:** Weighted demographic profile of the sample, by employment status: CCHS (2000–2014).

	Unemployed							Employed						
	2000/01	2003	2005	2007/08	2009/10	2011/12	2013/14	2000/01	2003	2005	2007/08	2009/10	2011/12	2013/14
Number of Observations	2724	2769	2801	2540	3089	2949	2763	51574	48229	48198	47415	41946	41038	39845
Age														
18–24	21.1%	18.8%	20.8%	18.3%	18.5%	19.5%	18.9%	9.7%	8.6%	8.7%	8.7%	8.3%	7.7%	8.2%
25–34	22.9%	26.3%	22.6%	25.0%	21.7%	21.1%	23.9%	23.4%	22.5%	22.0%	22.8%	21.7%	22.2%	22.4%
35–44	29.2%	26.1%	27.1%	21.4%	25.8%	20.0%	21.0%	31.5%	31.0%	29.2%	26.8%	25.8%	25.1%	24.7%
45–54	20.2%	19.9%	19.5%	21.2%	22.1%	23.7%	21.4%	25.7%	26.3%	27.8%	27.9%	29.0%	27.8%	26.6%
55–64	6.6%	8.9%	10.0%	14.2%	11.8%	15.7%	14.8%	9.7%	11.6%	12.4%	13.7%	15.3%	17.2%	18.0%
Sex														
Male	54.7%	56.6%	51.4%	56.0%	55.3%	54.6%	54.4%	59.8%	59.6%	59.5%	58.7%	57.6%	58.2%	57.8%
Female	45.3%	43.4%	48.6%	44.0%	44.7%	45.4%	45.6%	40.2%	40.4%	40.5%	41.3%	42.4%	41.8%	42.2%
Marital Status														
Couple	48.2%	51.4%	52.0%	53.3%	49.1%	46.7%	47.4%	69.0%	69.6%	70.5%	67.5%	69.2%	68.4%	68.0%
Single	38.8%	38.7%	37.0%	36.9%	37.4%	41.7%	42.5%	22.0%	21.5%	21.0%	23.0%	21.4%	21.9%	22.6%
Widowed or divorced	12.9%	9.8%	11.0%	9.8%	13.3%	11.3%	9.8%	9.0%	8.8%	8.4%	9.4%	9.3%	9.5%	9.3%
Missing	0.1%	0.0%	0.0%	0.1%	0.2%	0.4%	0.3%	0.1%	0.2%	0.1%	0.1%	0.1%	0.2%	0.1%
Children														
None	37.9%	38.9%	37.6%	39.3%	41.7%	41.6%	41.6%	39.1%	38.9%	39.2%	43.0%	43.8%	44.1%	43.0%
One or more	61.0%	59.4%	61.8%	59.7%	58.1%	57.7%	57.2%	59.9%	60.1%	60.0%	56.3%	55.5%	55.3%	56.5%
Missing	1.1%	1.6%	0.7%	1.0%	0.2%	0.7%	1.2%	1.0%	1.0%	0.8%	0.7%	0.7%	0.6%	0.5%
Race														
White	78.5%	75.0%	73.0%	70.0%	70.0%	67.8%	65.9%	85.9%	84.6%	83.1%	80.0%	79.8%	77.6%	75.7%
Black	3.2%	2.5%	3.0%	3.1%	4.1%	5.7%	5.2%	1.6%	1.7%	1.8%	2.4%	2.3%	2.1%	2.7%
Aboriginal	3.5%	2.9%	4.4%	7.1%	4.6%	5.9%	6.0%	1.1%	1.3%	2.0%	2.8%	2.9%	3.2%	3.1%
Asian	12.3%	14.4%	13.3%	15.1%	16.4%	16.8%	16.8%	8.9%	9.3%	9.8%	11.7%	11.8%	13.1%	14.1%
Multiple or other	2.4%	4.8%	5.7%	4.4%	4.1%	2.3%	5.2%	2.3%	3.0%	2.8%	2.5%	2.8%	3.2%	3.7%
Missing	0.1%	0.3%	0.6%	0.3%	0.8%	1.6%	0.9%	0.1%	0.1%	0.4%	0.5%	0.4%	0.8%	0.8%
Immigrant Status														
Non-immigrant	77.1%	72.6%	73.6%	73.2%	71.7%	72.3%	70.3%	79.5%	78.9%	78.6%	76.9%	76.6%	76.3%	74.9%
Immigrant: <15 years	12.9%	15.9%	14.3%	15.6%	13.8%	12.7%	16.2%	8.4%	8.2%	8.0%	9.0%	9.4%	9.3%	10.1%
Immigrant: 15+ years	8.6%	10.8%	10.8%	9.7%	12.5%	13.6%	12.2%	11.8%	12.7%	13.0%	13.6%	13.4%	13.2%	13.7%
Missing	1.5%	0.6%	1.3%	1.5%	2.0%	1.5%	1.4%	0.3%	0.3%	0.4%	0.4%	0.6%	1.2%	1.3%
Region														
Atlantic Canada	10.8%	10.8%	9.0%	10.0%	8.5%	9.0%	9.0%	7.3%	7.2%	7.1%	6.9%	6.9%	6.6%	6.5%
Central Canada	63.2%	62.8%	65.6%	66.8%	62.3%	64.4%	65.4%	63.0%	62.1%	62.1%	61.6%	61.5%	61.0%	60.2%
Western Canada	26.0%	26.4%	25.3%	23.3%	29.2%	26.6%	25.5%	29.7%	30.7%	30.7%	31.5%	31.6%	32.4%	33.3%
Area														
Urban	82.7%	81.1%	82.6%	83.1%	82.6%	84.3%	85.4%	82.0%	82.0%	82.4%	82.8%	82.7%	82.8%	82.4%
Rural	17.3%	18.9%	17.4%	16.9%	17.4%	15.7%	14.6%	18.0%	18.0%	17.6%	17.2%	17.3%	17.2%	17.6%

**Table 4:** Weighted socioeconomic profile of the sample, by employment status: CCHS (2000–2014).

	Unemployed							Employed						
	2000/01	2003	2005	2007/08	2009/10	2011/12	2013/14	2000/01	2003	2005	2007/08	2009/10	2011/12	2013/14
Number of Observations	2724	2769	2801	2540	3089	2949	2763	51574	48229	48198	47415	41946	41038	39845
Education														
Post-secondary	16.5%	20.1%	20.2%	18.8%	20.1%	17.6%	17.6%	21.2%	23.2%	25.0%	26.0%	27.9%	23.1%	23.2%
Some post-secondary	29.5%	32.8%	35.4%	35.6%	37.6%	41.1%	35.1%	35.3%	36.8%	41.1%	41.2%	40.9%	42.4%	39.7%
Secondary	28.5%	26.6%	25.9%	27.3%	24.7%	23.3%	32.6%	28.5%	27.1%	22.9%	23.1%	22.3%	26.4%	29.6%
Less than secondary	24.8%	19.5%	18.2%	18.2%	17.0%	17.2%	14.2%	14.5%	11.7%	10.6%	9.4%	8.6%	7.5%	7.0%
Missing	0.7%	1.0%	0.3%	0.1%	0.7%	0.7%	0.4%	0.5%	1.2%	0.4%	0.2%	0.3%	0.7%	0.5%
Income Decile														
1st	24.1%	20.1%	23.2%	24.8%	23.9%	24.4%	21.0%	3.9%	4.1%	4.4%	4.3%	4.0%	3.8%	3.9%
2nd	13.0%	11.4%	12.9%	14.4%	14.3%	13.2%	16.8%	5.3%	5.5%	5.8%	5.8%	5.4%	5.6%	5.5%
3rd	10.1%	10.1%	11.6%	11.4%	10.6%	13.5%	11.5%	7.0%	6.9%	7.0%	7.3%	7.3%	7.6%	7.8%
4th	8.5%	8.0%	10.0%	9.9%	8.6%	9.5%	9.5%	8.2%	8.5%	8.6%	9.0%	9.2%	8.7%	8.7%
5th	8.3%	10.2%	9.6%	8.4%	10.7%	9.3%	10.4%	9.8%	9.5%	10.3%	10.1%	9.8%	9.5%	10.0%
6th	5.8%	6.5%	8.4%	7.3%	8.6%	6.1%	8.6%	10.0%	9.9%	10.6%	11.0%	11.1%	10.7%	10.9%
7th	5.2%	5.2%	7.8%	7.6%	7.7%	7.7%	7.1%	11.0%	10.5%	12.0%	11.4%	12.4%	12.7%	12.4%
8th	4.7%	4.9%	6.0%	6.3%	6.5%	6.1%	5.6%	11.9%	11.2%	12.6%	13.1%	13.0%	12.7%	13.0%
9th	5.1%	4.3%	5.9%	5.5%	4.6%	4.8%	4.6%	12.0%	12.3%	14.3%	13.4%	13.5%	14.0%	13.7%
10th	5.1%	4.5%	4.5%	4.4%	4.5%	5.4%	4.9%	13.0%	12.9%	14.4%	14.6%	14.3%	14.5%	14.3%
Missing	10.2%	14.8%	0.0%	0.0%	0.0%	0.0%	0.0%	7.9%	8.7%	0.0%	0.0%	0.0%	0.0%	0.0%
Home Ownership														
Renter	50.4%	40.5%	41.1%	45.1%	43.2%	43.4%	47.1%	27.9%	23.0%	21.8%	24.1%	23.5%	24.8%	25.4%
Owner	49.3%	59.2%	58.3%	54.9%	56.5%	56.0%	52.6%	71.9%	76.7%	77.4%	75.2%	76.3%	74.8%	74.2%
Missing	0.4%	0.3%	0.6%	0.0%	0.3%	0.6%	0.3%	0.2%	0.2%	0.8%	0.6%	0.2%	0.4%	0.4%
Employment Insurance														
Not receiving	68.2%	56.7%	63.3%	67.3%	54.3%	63.3%	62.6%							
Receiving	29.8%	40.0%	32.8%	29.6%	39.3%	30.5%	31.6%							
Missing	2.0%	3.3%	3.9%	3.2%	6.4%	6.2%	5.8%							
Social Assistance														
Not receiving	75.1%	82.1%	79.7%	81.5%	78.5%	76.2%	78.5%							
Receiving	22.8%	14.6%	16.4%	15.3%	15.1%	17.6%	15.7%							
Missing	2.0%	3.3%	3.9%	3.2%	6.4%	6.2%	5.8%							

**Table 5:** Weighted proximal risk profile of the sample, by employment status: CCHS (2000–2014).

	Unemployed							Employed						
	2000/01	2003	2005	2007/08	2009/10	2011/12	2013/14	2000/01	2003	2005	2007/08	2009/10	2011/12	2013/14
Number of Observations	2724	2769	2801	2540	3089	2949	2763	51574	48229	48198	47415	41946	41038	39845
Self-Rated Stress														
Low	72.1%	74.5%	73.1%	74.8%	72.2%	75.5%	75.2%	69.3%	69.9%	71.6%	72.9%	71.4%	71.6%	71.9%
High	27.8%	25.3%	26.4%	24.6%	27.4%	24.1%	24.7%	30.6%	29.9%	28.1%	26.8%	28.5%	28.1%	27.9%
Missing	0.1%	0.5%	0.4%	0.6%	0.5%	0.3%	0.1%	0.1%	0.2%	0.3%	0.3%	0.1%	0.3%	0.2%
Chronic Conditions														
No	81.5%	82.3%	83.5%	82.5%	83.9%	81.2%	82.3%	87.3%	86.7%	86.4%	81.7%	86.1%	85.7%	85.7%
Yes	18.2%	17.3%	16.3%	17.1%	15.6%	18.5%	17.1%	12.7%	13.1%	13.4%	18.1%	13.7%	14.0%	14.1%
Missing	0.3%	0.5%	0.2%	0.4%	0.5%	0.2%	0.6%	0.1%	0.2%	0.2%	0.2%	0.2%	0.3%	0.2%
Hypertension														
No	92.1%	90.6%	91.0%	87.5%	88.1%	87.8%	86.4%	91.8%	90.3%	89.6%	89.0%	88.4%	87.8%	87.9%
Yes	7.7%	8.8%	8.7%	12.2%	11.7%	11.8%	12.1%	8.0%	9.6%	10.2%	10.7%	11.4%	11.9%	11.9%
Missing	0.2%	0.7%	0.3%	0.3%	0.1%	0.4%	1.5%	0.1%	0.2%	0.2%	0.3%	0.2%	0.3%	0.2%
Obesity														
No	82.1%	82.2%	81.1%	77.2%	78.9%	76.2%	77.2%	81.6%	82.2%	81.3%	79.7%	79.1%	78.0%	77.3%
Yes	14.9%	14.7%	16.1%	18.0%	17.8%	20.2%	18.6%	14.4%	15.1%	16.2%	16.5%	17.7%	18.6%	19.6%
Missing	3.0%	3.1%	2.8%	4.8%	3.3%	3.6%	4.1%	3.9%	2.7%	2.4%	3.8%	3.2%	3.4%	3.1%
Smoking														
Never smoked	26.5%	29.3%	31.5%	30.5%	33.7%	31.6%	32.5%	31.3%	30.7%	33.0%	35.0%	37.1%	37.1%	39.3%
Former smoker	26.8%	33.3%	29.9%	30.9%	26.7%	30.6%	30.5%	38.1%	41.2%	40.6%	38.8%	39.2%	39.0%	38.6%
Current smoker	46.6%	37.4%	38.5%	38.6%	39.5%	37.6%	36.9%	30.4%	27.9%	26.3%	26.1%	23.7%	23.7%	21.9%
Missing	0.1%	0.0%	0.0%	0.1%	0.2%	0.2%	0.0%	0.2%	0.2%	0.1%	0.1%	0.1%	0.2%	0.1%
Drinking														
Non-drinker	19.5%	19.7%	18.9%	20.4%	20.3%	20.2%	20.5%	12.9%	12.4%	12.5%	12.9%	13.5%	13.9%	14.5%
Moderate drinker	55.0%	56.6%	59.4%	55.4%	54.3%	54.3%	54.3%	65.8%	64.9%	64.0%	63.9%	63.1%	61.3%	60.1%
Binge drinker	25.0%	23.3%	21.4%	23.6%	24.9%	25.0%	24.1%	20.8%	22.3%	22.9%	22.7%	23.0%	24.3%	24.8%
Missing	0.5%	0.4%	0.4%	0.6%	0.5%	0.6%	1.2%	0.5%	0.5%	0.5%	0.5%	0.5%	0.5%	0.6%
Physical Activity														
Sufficiently active	22.7%	29.5%	28.8%	29.2%	29.3%	32.2%	33.8%	18.0%	23.8%	23.6%	22.9%	25.5%	26.1%	27.0%
Somewhat active	22.7%	24.8%	25.1%	23.7%	24.6%	25.7%	22.9%	21.7%	25.5%	25.2%	24.7%	25.1%	26.0%	26.0%
Inactive	49.4%	43.8%	45.5%	45.4%	45.4%	40.9%	42.6%	52.6%	49.6%	50.5%	51.1%	48.8%	47.4%	46.7%
Missing	5.1%	1.9%	0.6%	1.7%	0.7%	1.2%	0.7%	7.7%	1.2%	0.8%	1.3%	0.6%	0.5%	0.3%

**Fig 1:**
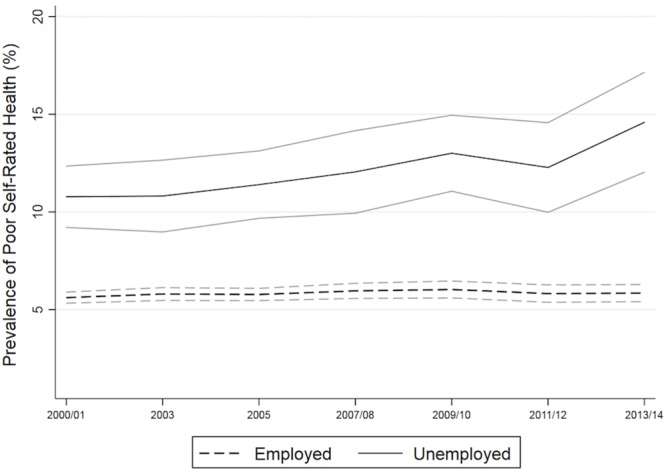
Weighted prevalence of poor self-rated health, by employment status: CCHS (2000–2014).

Fig 1 depicts trends in the unadjusted prevalence of poor self-rated health over the study period, separated by employment status. As expected, unemployed individuals reported consistently worse levels of poor self-rated health than their employed counterparts. Between 2000/2001 and 2013/2014, rates of poor self-rated health were relatively stable among the employed, hovering from year to year between 5.6% and 6.0%. By contrast, the prevalence of poor self-rated health increased from 10.8% to 14.6% among the unemployed. Due to these diverging trends, absolute unemployment-related inequalities in poor self-rated health increased from 5.2 percentage points to 8.7 percentage points over the study period.

### Decomposing change in the self-rated health of the unemployed

We decomposed change over time in the self-rated health of unemployed Canadians (Table 6). Between 2000/2001 and 2013/2014, the prevalence of poor self-rated health in this group increased by 3.8 percentage points. The decomposition results suggest that demographic, socioeconomic, and proximal risk factors included in our study fail to account for this increase. Endowing those who were unemployed in 2013/2014 with the full predictor profile of their counterparts in 2000/2001 was predicted to widen the gap by a further 0.5 percentage points (SE: 0.006, p = 0.425). The demographic endowment was predicted to narrow the gap by 1.0 percentage points (SE: 0.005, p = 0.033), while the socioeconomic and proximal endowments were predicted to widen the gap by 1.1 percentage points (SE: 0.004, p = 0.012) and 0.4 percentage points (SE: 0.004, p = 0.398), respectively. Because the deficits induced by the socioeconomic and proximal endowments were larger than the favourable returns from the demographic endowment, the decomposition model predicted a larger residual difference than that which was originally observed (4.3 percentage points versus 3.8 percentage points).

**Table 6:** Decomposition of poor self-rated health: Unemployed 2013/2014 versus unemployed 2000/2001.

Unemployed (2013/2014)	14.6%	N = 2763	
Unemployed (2000/2001)	10.8%	N = 2724	
	Estimate	SE	p
**Difference**			
Total	0.038	0.015	0.013
Explained	-0.005	0.006	0.425
Unexplained	0.043		
**Decomposition**			
*Demographic*			
Age	0.003	0.003	0.253
Sex	0.000	0.000	0.924
Marital Status	0.001	0.001	0.636
Children	0.004	0.002	0.025
Race	0.004	0.004	0.320
Immigrant Status	-0.002	0.003	0.537
Region	0.000	0.001	0.938
Urban/Rural	-0.001	0.001	0.357
Total	0.010	0.005	0.033
*Socioeconomic*			
Education	-0.004	0.003	0.168
Income	-0.002	0.004	0.522
Home Ownership	0.000	0.001	0.959
Employment Insurance	-0.002	0.001	0.080
Social Assistance	-0.003	0.002	0.188
Total	-0.011	0.004	0.012
*Proximal*			
Stress	-0.003	0.002	0.081
Chronic Conditions	0.001	0.002	0.479
Hypertension	0.006	0.002	0.017
Obesity	0.000	0.001	0.674
Smoking	-0.003	0.002	0.065
Drinking	-0.001	0.001	0.295
Physical Activity	-0.003	0.002	0.140
Total	-0.004	0.004	0.398

Note: Estimates and standard errors (SE) are generated from 1000 bootstrap samples.

### Decomposing self-rated health inequalities between the employed and the unemployed

We also decomposed self-rated health inequalities between employed and unemployed Canadians at each separate point in time (Table 7). We observed large and positive endowment terms across all time points, though the portion of unemployment-related health inequalities accounted for by the full set of predictors varied considerably from one time point to another. For example, they accounted for 3.6 of the 5.2 percentage point gap in 2000/2001 (SE: 0.003, p<0.001) and 4.0 of the 8.7 percentage point gap in 2013/2014 (SE: 0.005, p<0.001). Demographic factors appeared to play very little role in this story, as they were consistently associated with small or negligible individual and overall endowment terms. By contrast, we observed large socioeconomic endowment terms at each point in time. For example, endowing unemployed individuals with the more favourable socioeconomic profile of their employed counterparts was predicted to narrow the gap in poor self-rated health by 2.3 percentage points in 2000/2001 (SE: 0.003, p<0.001) and 2.8 percentage points in 2013/2014 (SE: 0.006, p<0.001). Finally, the endowment of proximal risk profiles produced moderately sized estimates. For example, proximal endowments were predicted to close the gap by 1.3 percentage points in 2000/2001 (SE: 0.001, p<0.001) and 0.8 percentage points in 2013/2014 (SE: 0.003, p = 0.003). Notably, closing the gap in the demographic, socioeconomic, and proximal characteristics of the employed and unemployed subgroups was not sufficient to eliminate self-rated health inequalities between them, as evidenced by the large unexplained residual terms reported at each point in time.

**Table 7:** Decomposition of poor self-rated health: Unemployed versus employed.

	2000/01			2003			2005			2007/08			2009/10			2011/12			2013/14		
Unemployed	10.8%	N = 2724		10.8%	N = 2769		11.4%	N = 2801		12.0%	N = 2540		13.0%	N = 3089		12.3%	N = 2949		14.6%	N = 2763	
Employed	5.6%	N = 51574		5.8%	N = 48229		5.8%	N = 48198		6.0%	N = 47415		6.0%	N = 41946		5.8%	N = 41038		5.8%	N = 39845	
	Estimate	SE	p	Estimate	SE	p	Estimate	SE	p	Estimate	SE	p	Estimate	SE	p	Estimate	SE	p	Estimate	SE	p
**Difference**																					
Total	0.052	0.008	<0.001	0.050	0.001	<0.001	0.056	0.009	<0.001	0.060	0.011	<0.001	0.070	0.010	<0.001	0.065	0.012	<0.001	0.087	0.013	<0.001
Explained	0.036	0.003	<0.001	0.027	0.003	<0.001	0.025	0.003	<0.001	0.036	0.004	<0.001	0.034	0.004	<0.001	0.044	0.004	<0.001	0.040	0.005	<0.001
Unexplained	0.016			0.024			0.031			0.024			0.036			0.021			0.047		
**Decomposition**																					
*Demographic*																					
Age	-0.004	0.001	<0.001	-0.001	0.001	0.232	-0.002	0.001	0.054	-0.003	0.001	0.018	0.002	0.003	0.592	-0.001	0.001	0.425	-0.001	0.002	0.732
Sex	0.000	0.000	0.620	0.000	0.000	0.132	-0.001	0.000	0.106	0.000	0.000	0.409	0.000	0.000	0.809	0.000	0.000	0.962	0.000	0.000	0.263
Marital Status	0.003	0.001	0.002	0.001	0.001	0.265	0.002	0.001	0.036	0.004	0.001	0.002	0.001	0.004	0.827	0.005	0.002	0.005	0.004	0.002	0.107
Children	0.000	0.000	0.183	0.000	0.000	0.717	0.000	0.000	0.674	0.000	0.000	0.667	0.000	0.000	0.696	0.000	0.000	0.884	0.000	0.000	0.340
Race	0.001	0.001	0.223	0.002	0.001	0.039	0.001	0.001	0.308	0.002	0.001	0.058	-0.001	0.003	0.693	0.002	0.001	0.120	0.002	0.001	0.105
Immigrant Status	-0.001	0.000	0.171	0.000	0.001	0.674	-0.001	0.001	0.434	-0.002	0.001	0.084	0.000	0.002	0.838	0.000	0.001	0.984	-0.001	0.001	0.123
Region	0.000	0.000	0.523	0.000	0.000	0.103	-0.001	0.000	0.002	-0.002	0.001	0.004	-0.001	0.001	0.481	-0.002	0.001	0.005	-0.001	0.001	0.383
Urban/Rural	0.000	0.000	0.390	0.000	0.000	0.519	0.000	0.000	0.895	0.000	0.000	0.941	0.000	0.000	0.984	0.000	0.000	0.955	0.000	0.000	0.132
Total	0.000	0.001	0.752	0.003	0.002	0.114	-0.002	0.002	0.329	-0.001	0.002	0.615	0.000	0.005	0.974	0.004	0.002	0.136	0.003	0.003	0.232
*Socioeconomic*																					
Education	0.006	0.001	<0.001	0.005	0.001	<0.001	0.005	0.001	<0.001	0.006	0.001	<0.001	0.007	0.001	<0.001	0.005	0.001	<0.001	0.003	0.001	0.003
Income	0.015	0.002	<0.001	0.012	0.003	<0.001	0.013	0.003	<0.001	0.019	0.004	0.000	0.015	0.004	<0.001	0.021	0.005	<0.001	0.023	0.005	<0.001
Home Ownership	0.002	0.001	0.078	0.000	0.001	0.791	0.000	0.001	0.925	0.001	0.002	0.671	0.004	0.004	0.314	0.003	0.002	0.113	0.001	0.002	0.691
Total	0.023	0.003	<0.001	0.017	0.003	<0.001	0.019	0.003	<0.001	0.026	0.004	<0.001	0.026	0.005	<0.001	0.028	0.005	<0.001	0.028	0.006	<0.001
*Proximal*																					
Stress	-0.001	0.000	0.063	-0.002	0.000	<0.001	0.001	0.000	0.030	0.000	0.000	0.618	0.001	0.002	0.492	-0.002	0.001	0.004	0.000	0.001	0.660
Chronic Conditions	0.009	0.001	<0.001	0.007	0.001	<0.001	0.006	0.001	<0.001	0.006	0.001	<0.001	0.006	0.001	<0.001	0.008	0.001	<0.001	0.007	0.001	0.000
Hypertension	-0.001	0.000	0.015	-0.001	0.000	0.034	-0.002	0.000	<0.001	0.001	0.000	0.007	0.000	0.001	0.630	0.001	0.000	0.066	0.000	0.001	0.677
Obesity	0.001	0.000	0.085	0.000	0.000	0.454	0.000	0.000	0.591	0.001	0.000	0.024	0.000	0.001	0.807	0.001	0.001	0.084	-0.001	0.001	0.077
Smoking	0.005	0.001	<0.001	0.003	0.001	<0.001	0.006	0.001	<0.001	0.005	0.001	<0.001	0.003	0.003	0.324	0.005	0.001	<0.001	0.007	0.002	<0.001
Drinking	0.002	0.001	<0.001	0.002	0.001	0.002	0.002	0.000	<0.001	0.002	0.001	0.002	0.001	0.001	0.718	0.001	0.001	0.153	0.000	0.001	0.995
Physical Activity	-0.003	0.001	<0.001	-0.003	0.001	<0.001	-0.003	0.001	<0.001	-0.004	0.001	<0.001	-0.003	0.001	<0.001	-0.004	0.001	<0.001	-0.004	0.001	<0.001
Total	0.013	0.001	<0.001	0.007	0.001	<0.001	0.008	0.001	<0.001	0.011	0.002	<0.001	0.008	0.005	0.069	0.012	0.002	<0.001	0.008	0.003	0.003

Note: Estimates and standard errors (SE) are generated from 1000 bootstrap samples.

As noted above, the absolute gap in self-rated health between the employed and unemployed subgroups widened over the study period from 5.2% to 8.7%. However, we did not observe commensurate growth in the explanatory capacity of our predictors. The demographic and proximal endowment terms did not grow larger over time. In fact, the proximal endowment term decreased from 1.3 percentage points in 2000/2001 (SE: 0.001, p<0.001) down to 0.8 percentage points in 2013/2014 (SE: 0.003, p = 0.003). Our results provide some indication of a small absolute increase in the size of the socioeconomic endowment terms, from 2.3% percentage points in 2000/2001 (SE: 0.003, p<0.001) to 2.8 percentage points in 2013/2014 (SE: 0.006, p<0.001). This increase appears to be entirely attributable to household income, whose absolute contribution as an individual predictor increased from 1.5 percentage points in 2000/2001 (SE: 0.002, p<0.001) to 2.3 percentage points in 2013/2014 (SE: 0.005, p<0.001). Overall, the predictors accounted for a smaller portion of observed inequalities in 2013/2014 than in 2000/2001. Consequently, the residual term was larger in 2013/2014. Whereas the unexplained difference was 1.6 percentage points in 2000/2001, it was 4.7 percentage points in 2013/2014.”

## Discussion

We used population-based data from a repeated cross-sectional survey to examine changing patterns of self-rated health among employed and unemployed Canadians from 2000 to 2014. Our results indicate that relative and absolute inequalities in poor self-rated health increased between the two groups over the study period. These findings mirror those reported in recent studies that have also documented widening unemployment-related health inequalities in Germany, Sweden, and the United Kingdom [12–14]. Unexpectedly, in our decomposition of these trends, demographic, socioeconomic, and proximal risk factors did not explain the growing self-rated health gap. On the contrary, the extent to which they accounted for unemployment-related health inequalities declined over time. As a result, the unexplained portion of the gap grew from 1.6 percentage points in 2000/2001 to 4.7 percentage points in 2013/2014.

In the introduction of our study, we presented several candidate explanations for widening unemployment-related health inequalities. The mathematical artifact hypothesis maintains that relative inequalities in the health status of the employed and the unemployed may have a tendency to grow as a result of overall improvements in the absolute prevalence of adverse health outcomes [16–20]. However, we found no evidence of an overall decline in the prevalence of poor self-rated health. Among the employed, for example, the prevalence of poor self-rated health did not vary substantially from one time point to the next. Furthermore, the self-rated health gap between the employed and the unemployed grew in both relative and absolute terms. Thus, it is unlikely that our findings are merely an artifact of measurement.

A second view of the problem suggests that unemployment-related health inequalities may be widening due to increasing opportunities for indirect social selection on the basis of personal characteristics such as intelligence and cognitive ability, which may predict both the health and labour market outcomes of individuals [22–28]. As noted earlier, this argument is premised on the assumption that advanced capitalist societies have become more meritocratic over time. However, evidence from the broader literature indicates that rates of social mobility in Canada have declined over the past few decades [87]. Moreover, levels of educational attainment among unemployed increased substantially over the study period. Whereas 25.0% of unemployed Canadians reported having less than a high school degree in 2000/2001, only 14.2% of unemployed Canadians belonged to this category in 2013/2014. In other words, our evidence suggests that unemployed Canadians become a less negatively selected group over time. Although we were unable to test the indirect selection hypothesis directly, these empirical developments are at odds with its theoretical expectations.

The third hypothesis posits that unemployment-related health inequalities may be widening due to widening inequalities in the uneven distribution of proximal risk factors between the employed and the unemployed [31–38]. Although they went part of the way in explaining why unemployed individuals reported worse levels of self-rated health than their employed counterparts, trends in the distribution of these proximal risk factors explained neither the increasing prevalence of poor self-rated health among unemployed individuals nor the growing health gap between employed and unemployed individuals. These findings may reflect the fact that we did not capture the full set of proximal mechanisms linking unemployment and health, including those whose salience may have increased over time (e.g. psychosocial factors, dietary behaviours, and food insecurity). It may also be the case that, for reasons not yet understood, the adverse returns to specific exposures have increased over time, such that widening unemployment-related health inequalities do not reflect changes in the distribution of proximal risk factors but rather changes in the strength of their association with health. Prior research suggests that the association between a given risk factor and health can vary over time [88], and that this variation can contribute to widening health inequalities between socioeconomic groups[89]. For example, there is evidence that the widening mortality gap between educational groups in the United States is not a result of changes in the distribution of risk factors such as smoking and obesity but, rather, is explained by the increasing severity of the mortality consequences associated with these risk factors [90]. Because differences in the effects of predictors are hidden in the residual component of our decomposition, we were not able to quantify the contribution of this heterogeneity to the growing gap.

Our final hypothesis suggests that unemployment-related health inequalities may be worsening due to increasing inequalities in the underlying social determinants of health [12–14,44–49]. While socioeconomic factors such as income, education, and home ownership provided the strongest explanation for self-rated health inequalities between employed and unemployed Canadians, they accounted for only a marginal portion of the growth observed in the magnitude of these inequalities over time. Furthermore, they were incapable of accounting for the increasing prevalence of poor self-rated health among the unemployed. These results could reflect the fact that key factors such as wealth, lifecourse socioeconomic status, and financial strain—factors which are known to differ substantially between the employed and the unemployed—were not reported in the CCHS and therefore could not be included in our analysis. Indeed, a growing body of research suggests that, variables such as financial strain exhibit an independent association with health, over and above conventional measures of income [48,91,92]. Another possible explanation may be that markers of socioeconomic position are not equivalent over time. Rather, their meaning may change from one historical context to the next and, as a result, their association with health may also change over time. For example, given that the cost of housing has outpaced average earnings in Canada [93], it is possible that the relative disadvantage associated with renting as opposed to owning one’s home has increased over time. Similarly, given widening levels of income inequality, it may be the case that the extent of deprivation experienced by those in the lowest decile of earnings has increased in a manner that our categorical income variable is incapable of capturing. Put simply, whether an unemployed individual is as likely as before to fall into one or another socioeconomic category may matter less than changes over time in the magnitude of the effects associated with a given category. Indeed, in supplementary analyses of our data (not shown), we found evidence that the strength of the association between key socioeconomic factors (e.g. home ownership and income) and health grew substantially over the course of the study period. As noted above, this heterogeneity in effect sizes is hidden in the residual component of the decomposition and must therefore be investigated elsewhere.

The notion that the meaning of socioeconomic categories can change over time presents the possibility of a final and related explanation of our findings; namely, that the health status of employed and unemployed Canadians may be diverging as a result of changing macrosocial contexts whose underlying dynamics and consequences cannot be captured using routinely available survey data. From the broader literature, we know that similar socioeconomic experiences do not produce the same set of health outcomes from one national context to the next. For example, the magnitude of unemployment-related health inequalities varies considerably across countries [14,50,94,95]. These findings are thought to reflect the fact that structural factors such as policy environments (e.g. unemployment benefit systems) play a pivotal role in shaping the health gradient [96,97]. In a similar vein, contextual trends such as rising levels of income inequality, weakening social safety nets, and declining levels of social spending may be fundamental contributors to widening health inequalities, including those observed between the employed and the unemployed [6,7,12,14,77,98]. Unfortunately, due to the nature of our data, we were unable to directly quantify the contribution of these broader societal trends. Nevertheless, these developments are elsewhere understood as part of a broader neoliberal transformation of society whose implications for health are increasingly well-documented [99–102].

Our study has several limitations in addition to those mentioned above. First, like many of its peer nations, Canada experienced a recession between 2008 and 2010. Unemployment rates in Canada increased between 2000 and 2009, from 5.7% to 7.0%, then declined in a secular fashion to 5.8% in 2014 [103]. During this time, overall labour force participation rates remained stable, fluctuating between 66.0% and 67.6% [103]. In general, Canada experienced a shorter and milder recession than other advanced capitalist countries [104]. Nevertheless, it is possible that fluctuating rates of unemployment over the course of the study period biased our results [105]. However, an earlier Canadian study found that the association between unemployment and health did not vary according to local unemployment rates [106]. Moreover, unemployment rates were very similar at our first and final time points (i.e. approximately 6%). Thus, any resulting bias is not likely to have influenced our most important set of findings. Second, there is evidence that, over the study period, a growing proportion of jobless individuals became discouraged and gave up on actively seeking employment [107]. The increasing tendency for these discouraged workers to select out of unemployment and into inactivity may have biased our findings. However, because these individuals could not be identified in the CCHS, we were unable to investigate the impact of this potential selection problem on our results. Third, many of our measures, including our outcome of interest, relied on self-report and are therefore susceptible to corresponding biases. For example, there is some evidence of an interaction between socioeconomic status and the predictive validity of self-rated health, though findings on this issue are mixed [108]. In addition, the use of self-report in both the outcome and some predictor variables could have contributed to sole source bias [109]. Fourth, trends in unemployment-related health inequalities, as well as their underlying causes, may differ between men and women [13,110]. However, due to a limited sample of unemployed persons as well as the nature and number of the covariates included in our models, we lacked sufficient statistical power to conduct separate analyses for men and women. This is an important gap for future research to address. Fifth, risk factors such as smoking, binge drinking, and physical inactivity may precede and contribute to unemployment [111]. For example, there is literature suggesting that earlier binge drinking is as associated with later life socioeconomic adversity, including job loss [112–114]. Given the cross-sectional nature of our data, we were unable to account for this potential endogeneity problem.

Finally, as we have previously noted, a substantial portion of household income values were missing in 2000/2001 and 2003. Prior research suggests that individuals who withhold from reporting income values tend to have a worse socioeconomic profile relative to those who do report [115]. Indeed, our own supplementary analyses (not shown) revealed that respondents in the missing category reported lower levels of educational attainment and home ownership than their non-missing counterparts. Thus, we expect that the true distribution of income among those who were unemployed in 2000/2001 was worse than the distribution we observed and could endow to their counterparts in 2013/2014. Accordingly, we anticipate that the true explained component in Table 6 is even smaller (i.e. more negative) than that we would have reported in our results. In other words, the results we have reported are likely more conservative than those we would have reported in the absence of this missing information. Moreover, in Table 7, the results reported for 2000/2001 and 2003 are on par with those reported in neighboring years (e.g. 2005 and 2007/2008). Thus, again, we do not anticipate that missing income information caused any substantial bias.

Notwithstanding these limitations, there are important insights to be gained from our study. Most notably, the factors that are known to explain why the employed are healthier than their unemployed counterparts do not appear to explain why health inequalities between these two groups have widened over time [116]. While unemployed Canadians tended toward less favourable socioeconomic and proximal risk profiles, these individual-level predictors could not account for adverse trends in the relative or absolute health status of this group. These findings lend support to the notion, now common in the social epidemiological literature, that there are forces acting upon the health of populations over and above the set of individual-level attributes on which data are routinely collected [117–119]. The implication is that changing patterns of unemployment-related health inequality must be situated within the context of broader macrosocial trends such as widening income inequalities, declining social safety nets, and decreasing social spending. These higher-order phenomena are not always easily incorporated into the individual risk functions that prevail in contemporary epidemiologic research. Nevertheless, if our results are any indication, making sense of widening health inequalities between the employed and the unemployed may depend on our willingness to appropriately measure and model these underlying macrosocial trends.
